# A Secure Multi-Layer Edge-Based Sensor Architecture for Building-Level Disaster Monitoring and Decision Support

**DOI:** 10.3390/s26144531

**Published:** 2026-07-17

**Authors:** Kerem Erzurumlu, Kenan Rıfat Erzurumlu

**Affiliations:** 1 Department of Computer Technologies, Vocational School of Technical Sciences, Ordu University, 52200 Ordu, Turkey; 2 Department of Electrical and Electronics Engineering, Faculty of Engineering, Ege University, 35040 Izmir, Turkey; 05230000698@ogrenci.ege.edu.tr

**Keywords:** disaster management, early warning system, building monitoring, edge computing, central monitoring system, response prioritization

## Abstract

Natural and human-induced disasters can cause significant loss of life and property, particularly at the building level, highlighting the need for effective early detection, real-time monitoring, and rapid post-disaster response. Current disaster management approaches largely rely on citizen reports and manual observations, which may lead to delays and inefficient resource allocation, especially in large-scale events. This study proposes a secure, modular, multi-layer disaster monitoring and decision-support architecture that integrates sensor-based building-edge monitoring units deployed at both the building and apartment levels with a central emergency monitoring system. The architecture comprises three main layers: edge sensing, secure cellular communication, and central decision-making. Building-edge monitoring units collect data related to structural motion and inclination indicators, fire, flooding, and gas leaks, perform preliminary processing, and transmit aggregated data securely to the central system. Communication security is ensured through a certificate-based authentication mechanism supported by a dedicated certificate authority, reducing the risk of unauthorized access and fraudulent data injection. The central system performs automated event detection and separately evaluates physical building condition and communication status, enabling prioritized response planning. To evaluate feasibility, a two-building prototype was implemented and tested through scenario-based experiments involving two independently operating building-edge monitoring units connected to the same central monitoring system. The prototype demonstrated concurrent secure data acquisition and central aggregation from two buildings; however, district- and regional-scale performance requires further validation through larger-scale controlled load tests and field deployments. Under laboratory conditions, the prototype demonstrated sensor-data acquisition, authenticated transmission, and centralized event classification. End-to-end latency and building-edge monitoring unit power consumption were also measured; however, the prototype was not validated under environmental conditions representative of real disasters. Overall, the findings suggest that sensor-based, secure, and centralized monitoring systems may complement traditional disaster management approaches.

## 1. Introduction

Natural and human-induced disasters remain a major global concern because they cause loss of life, economic damage, and infrastructure destruction. Events such as earthquakes, fires, floods, and gas leaks can cause substantial financial damage during the disaster itself and throughout the early warning and initial response phases. In particular, rapid urbanization and increased building density make the impact of disasters more pronounced at the building level and highlight the importance of local-level measures [1].

Disaster response processes rely largely on citizen reports, on-site observations, and manual reporting mechanisms. This approach may result in delays in information flow, incomplete or erroneous guidance, and the inefficient use of limited resources. This problem becomes particularly severe during large-scale disasters, when simultaneous impacts on numerous buildings and infrastructure elements can make it difficult to determine which areas require priority intervention. This situation highlights the need for systems that generate automated, continuous, and objective data in disaster management.

The continuity of communication infrastructure is critical for early detection and rapid response during disasters. Although fixed communication infrastructure and local networks may become unavailable because of physical damage or power outages, cellular communication may provide an alternative data-transmission path. However, its availability cannot be guaranteed during a disaster because congestion, base-station power loss, backhaul disruption, physical damage, and temporary coverage degradation may affect communication performance. During disasters, public authorities commonly recommend data-based communication because it may enable more efficient transmission of essential information than voice-based communication under congested cellular network conditions [2].

The consequences of disasters are often most evident at the building level. Situations involving structural motion or inclination, fire, flooding, or gas leaks within a building may produce measurable changes in physical and environmental parameters before they are noticed by occupants. Continuous sensor-based monitoring enables the early identification of these risks. However, evaluating individual building data in isolation is insufficient; effective disaster management requires the integrated analysis of data from multiple buildings within a centralized system.

Centralized monitoring and decision-support mechanisms play a critical role in determining post-disaster response priorities. In the aftermath of major disasters, separately assessing physical building condition, communication status, and response priority can facilitate the effective deployment of search-and-rescue and emergency response teams. The implementation of a data-driven and automated prioritization process may reduce response times, thus minimizing loss of life and property.

The widespread adoption of centralized systems has also raised concerns about security and data integrity. In disaster management, data originating from erroneous, fraudulent, or unauthorized sources may generate false alarms and incorrect response decisions. Therefore, communication between edge devices and the central monitoring system should not rely solely on encryption; the identity of all components must also be authenticated. Certificate-based authentication and dedicated certificate authorities reduce the risk of unauthorized devices connecting to the system.

This study proposes a multi-layer disaster-monitoring architecture that integrates sensor-based edge units capable of monitoring multiple disaster types at the building level with a centralized emergency monitoring center operating at the regional level. The proposed architecture supports secure data transmission over a cellular communication infrastructure and reduces the risk of fraudulent data and unauthorized edge access through certificate-based authentication. Moreover, the system provides a conceptual framework for post-disaster response prioritization by separately evaluating physical condition, communication status, and response priority.

Recent research has increasingly focused on integrating IoT-based sensing with edge and cloud computing for real-time structural health monitoring. Edge-side processing has been employed to reduce communication load, improve response time, and support continuous monitoring using low-cost and low-power sensor nodes. These implementations have also demonstrated the use of IoT sensors, digital signal processing, and edge-enabled architectures for vibration monitoring, damage assessment, and real-time visualization of structural behavior. Many existing studies focus primarily on structural health monitoring of individual structures, whereas secure multi-building data aggregation and post-disaster response prioritization remain less extensively studied.

Existing studies commonly examine IoT-based structural monitoring, edge-side processing, and secure sensor communication separately or within individual structures. Limited attention has been given to architectures that combine multi-hazard sensing, secure building-edge aggregation, communication-status assessment, and central response prioritization across multiple buildings. The main contribution of this study is the integration of building- and apartment-level multi-hazard sensing, rule-based edge processing, certificate-based mutual authentication, communication-status differentiation, and central response prioritization within a unified architecture demonstrated through a two-building proof of concept.

## 2. Materials and Methods

### 2.1. System Architecture

**Figure 1:**
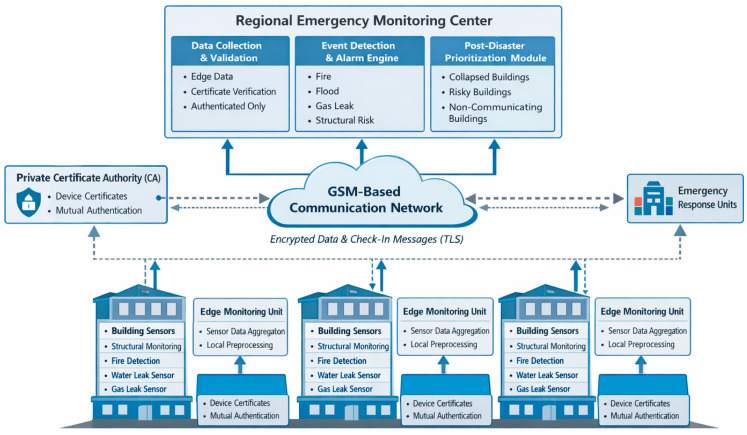
Overview of the proposed multi-layer emergency monitoring architecture.

The proposed system uses a multi-layer architecture comprising sensor-based edge monitoring units operating at the building and apartment levels and a central emergency monitoring center that processes data received from these units. As illustrated in Figure 1, the layered approach supports the secure transmission of building-level data to the central system and the operation of automated decision-support mechanisms before and after disasters [3].

The system comprises three primary layers: (i) the edge sensing layer, (ii) the secure communication layer, and (iii) the central decision and monitoring layer. Multi-layer architectures are widely used in sensor-based systems to separate data collection, transmission, and decision-making functions [4]. As shown in Figure 1, the architecture supports secure transmission to the central system by aggregating data from sensors deployed at the building and apartment levels.

The edge sensing layer comprises sensors installed in buildings and building-edge monitoring units that collect and process sensor data. Edge computing processes data in real time at the point of collection, thereby reducing the load on the central system [5]. Recent structural health monitoring studies have similarly employed edge computing to perform local signal processing, reduce transmitted data volume, and support real-time analysis on resource-constrained sensor nodes [6,7]. In the current prototype, edge-side processing is implemented through rule-based analytics rather than data-driven intelligent inference. The building-edge monitoring unit aggregates sensor measurements, evaluates them using building-specific reference values and baseline-relative changes, identifies abnormal conditions, and generates event-level outputs before transmission. Accordingly, the term edge computing in this study refers to local preprocessing, rule-based event detection, and communication-load reduction. This approach manages data volume at the edge layer and avoids the need for numerous sensors to communicate directly with the central system [8]. In this layer, data related to structural, environmental, and security risks are continuously monitored. While motion- and inclination-related structural indicators are monitored at the building level, events such as fire, flooding, and gas leaks can be detected at both building and apartment levels.

Data are transmitted between building-edge monitoring units and the central system through a cellular communication infrastructure. Cellular networks may provide greater flexibility and resilience than fixed infrastructure during large-scale disasters, although congestion, power failure, and infrastructure damage may still disrupt service [9]. This layer serves two purposes: data transmission and protection of communication confidentiality and integrity.

A certificate-based mutual authentication mechanism is a core component of the secure communication layer. The system therefore incorporates a dedicated certificate authority (CA). Communication is established only after both the building-edge monitoring units and the central system present certificates issued and validated by the CA. This approach reduces the risk of unauthorized devices sending fraudulent data by impersonating a legitimate building-edge monitoring unit, even if the addresses of communication channels or application interfaces are known. The certificate-based mutual authentication mechanism establishes the system’s chain of trust.

This public key infrastructure (PKI)-based approach reduces the risks of fraudulent data injection and unauthorized access by allowing only authenticated devices to transmit data to the system [10].

The certificate lifecycle includes device enrollment, issuance, renewal, revocation, replacement, and re-enrollment after credential compromise. Each building-edge monitoring unit stores its private key locally, and the key is never transmitted to the central system. The prototype did not include a dedicated secure element or hardware security module; therefore, protection against physical credential extraction was not evaluated at the hardware level. Certificates are renewed before expiration, and expired or revoked certificates are rejected during mutual authentication. If key compromise is suspected, the corresponding certificate is revoked, and a new key pair and certificate are issued after device re-enrollment. The central system maintains a locally cached trust and revocation record to support certificate validation during temporary network outages, and this information is synchronized when connectivity is restored.

The threat model considers device impersonation, unauthorized access, replay attacks, communication tampering, and compromised edge-device credentials. Each building-edge monitoring unit is assigned a unique public–private key pair and a device-specific certificate issued by a dedicated certificate authority. Mutual TLS authentication verifies both the building-edge monitoring unit and the central system before application data are exchanged. The prototype used a standard HTTPS/TLS configuration with certificate-chain validation, validity-period checks, device-identity verification, and certificate-revocation checks. The study did not introduce a new cryptographic protocol; therefore, the security configuration is reported at the architectural and functional levels. In the prototype, the Arduino Uno controlled message construction, timestamps, message identifiers, and application-level processing, whereas cellular connectivity and HTTPS/TLS transport were provided through the SIM7600G-H module under the control of the Arduino Uno.

TLS protects the confidentiality and integrity of the communications. Each application message also contains a timestamp, a unique message identifier, and the identity of the originating building-edge monitoring unit. The central system checks message freshness and rejects duplicate or previously processed identifiers to reduce replay risk. If an edge-device private key is lost or suspected to be compromised, the associated certificate is revoked and removed from the list of trusted devices.

Functional security checks were performed using invalid certificates, revoked device credentials, duplicate message identifiers, and altered messages. In each case, the central system rejected the message and recorded the event as a security violation.

The central emergency monitoring center constitutes the decision-support layer and performs automated event detection and response prioritization based on sensor data. Central decision support systems can support data integration and response coordination in disaster management [11,12].

Centralized analysis of sensor data enables the evaluation of event density and spatial distribution, particularly in multi-building scenarios [13]. This can inform post-disaster response prioritization based on automated and objective criteria.

The central emergency monitoring center is the primary decision-making layer where data from all building-edge monitoring units are collected, validated, and processed. As shown in Figure 1, this center comprises three core functional modules: data collection and validation, event detection and alarm generation, and post-disaster response prioritization.

The data collection and validation module accepts data exclusively from authenticated building-edge monitoring units and rejects unauthorized data entries through certificate checks. The incident detection and alarm generation module analyzes data related to fires, floods, gas leaks, and structural risks, and generates automated alerts. The post-disaster response prioritization module separately evaluates physical condition, communication status, and response priority after large-scale incidents to support the allocation of response teams.

The proposed architecture clearly separates sensing, communication, and decision-making functions, thereby supporting modular expansion and secure communication. Data collection and preprocessing at the edge layer reduce the load on the central system. The adoption of a secure cellular communication mechanism based on certificate authentication supports authenticated data transmission. The automated incident-detection and response-prioritization capabilities of the central monitoring center are intended to support faster and more objective decision-making than traditional approaches based on citizen reports.

### 2.2. Building-Edge Monitoring Unit

The building-edge monitoring unit is a core component of the edge sensing layer in the proposed architecture. The unit is a logical node that collects data from sensors deployed at both the building and apartment levels. The collected data are preprocessed and securely transmitted through an authenticated channel to the central emergency monitoring center. As shown in Figure 1, each building is assigned one building-edge monitoring unit, which aggregates data from the sensors installed in that building. Edge-based sensing approaches are used to reduce latency and communication load by processing data close to the source [5,6].

Sensors are widely used to monitor structural behavior, including the early detection of bending and foundation movement [14]. Recent studies have demonstrated that low-cost IoT-based sensor nodes can support real-time vibration monitoring, damage detection, and structural health assessment. Laboratory-scale implementations using microcontrollers and accelerometers have demonstrated the potential of cost-effective distributed monitoring approaches [15,16]. Sensor-based detection of environmental hazards, including fires, gas leaks, and flooding, is a key component of building safety and early warning systems [17].

**Figure 2:**
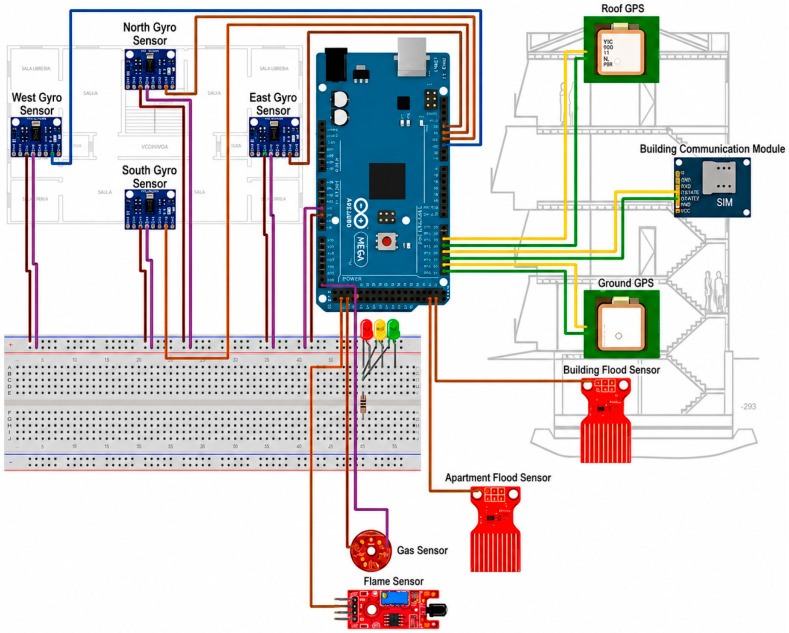
Representative sensor placement and connectivity within a single monitored building.

The building-edge monitoring unit manages data from multiple sensors used to monitor structural and environmental risks within a building. While structural-integrity data are evaluated at the building level, incidents such as fire, flooding, and gas leaks can be detected at both building and apartment levels. This approach distinguishes between common-area incidents and apartment-level incidents. Figure 2 illustrates representative sensor placement and connectivity within a single monitored building.

The data collected by apartment-level sensors are not transmitted directly to the central system; instead, they are aggregated by the building-edge monitoring unit. This reduces the data load on the communication infrastructure, even in buildings with high sensor density, and supports future system expansion.

The building-edge monitoring unit is not merely a passive raw-data forwarding component. It performs rule-based edge analytics, including sensor-data aggregation, baseline-relative evaluation, threshold-based event detection, persistence checks, and separation of routine check-in messages from abnormal-event notifications. These functions constitute local rule-based analytics but should not be interpreted as machine-learning-based edge intelligence. The sensor data are evaluated using building-specific reference values and threshold mechanisms rather than universal fixed thresholds. Each monitored building is assigned a configuration profile containing its structural system, number of stories, relevant geometric characteristics, sensor types and locations, and baseline measurements recorded during commissioning. Abnormal conditions are identified by evaluating both the absolute sensor value and its deviation from the building-specific baseline. This preprocessing serves two main functions: reducing the data volume and enabling the transmission of meaningful and interpretable information to the central system.

During commissioning, sensor measurements are collected under normal operating conditions over a predefined baseline-acquisition period. These measurements are used to determine the baseline, normal variation range, and measurement noise for each sensor. Warning and critical thresholds are then configured according to the building’s structural system, the location and orientation of the sensor, and the expected structural response at that measurement point. Threshold selection should be performed or approved by a qualified structural engineer in accordance with applicable structural design and assessment guidance. Accordingly, masonry, reinforced-concrete, and steel-frame buildings should not be evaluated using the same absolute motion or inclination threshold.

In the prototype, MPU6050 modules were installed on the four principal walls of each monitored building to detect changes in motion and inclination relative to the commissioning baseline. The accelerometer and gyroscope outputs were evaluated through rule-based comparison with the baseline to identify abnormal changes; they were not used to derive a direct engineering measurement of structural displacement. The NEO-6M module was used only to provide the building’s reference position and coarse elevation context. It was not used to measure small structural displacements. Accordingly, the prototype demonstrates the functional detection of motion- and inclination-related changes rather than metrologically validated structural deformation measurements.

Local preprocessing does not replace the central decision-making process; it is intended to identify potential risks at an early stage and enable the central system to respond more rapidly. Accordingly, the building-edge monitoring unit supports rather than replaces central decision-making.

The building-edge monitoring unit is a key component of the system’s security architecture. Each building-edge monitoring unit has a unique digital certificate issued by the system’s dedicated certificate authority. The certificate enables the central system to verify the unit’s identity, allowing only authorized edge devices to transmit data.

All data transmitted between the building-edge monitoring unit and the central monitoring center use encrypted communication channels. A certificate-based mutual authentication mechanism is used so that even if the addresses or application interfaces of the communication infrastructure are known, unauthorized devices cannot authenticate as legitimate building-edge monitoring units. This approach helps reduce the risk of false or unauthorized data entering the system following a disaster.

The building-edge monitoring unit is intended to operate before, during, and after disasters. Before a disaster, early indicators of structural motion, inclination changes, or environmental hazards are continuously monitored, and regular status updates (check-in messages) are sent to the central system. This approach supports the early identification of at-risk structures before a disaster occurs.

The proposed design includes provision for a 12 V backup battery to supply the controller, sensors, and cellular communication module during temporary mains-power interruptions. The required battery capacity and expected operating duration depend on the selected hardware, sensor load, cellular signal quality, transmission frequency, and power-management settings. Since long-duration battery-discharge testing was not performed in this study, backup operating time was not experimentally quantified. Accordingly, missing check-in messages after a power interruption indicate an unverified communication or power condition rather than direct evidence of structural damage.

**Figure 3:**
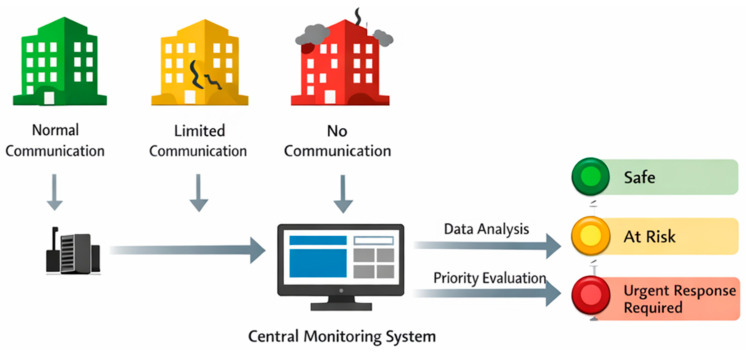
Conceptual relationship between building-edge communication status and post-disaster building assessment.

After a disaster, the building-edge monitoring unit continues transmitting check-in and sensor messages while communication remains available. However, the absence of data from a building-edge monitoring unit is not interpreted as evidence of structural damage. Following the first missed check-in, the central system assigns the provisional status “Communication Unverified” and initiates a zone-level communication assessment. Building-condition information and communication availability are maintained as separate decision variables so that an isolated communication failure does not directly trigger a physical-hazard classification or a search-and-rescue dispatch. Figure 3 summarizes the relationship between building-edge communication status and post-disaster assessment.

The building-edge monitoring unit is the primary building-level component of the proposed system. This approach, which aggregates data from multiple sensors into a single logical point, improves the use of the communication infrastructure and reduces the number of direct connections to the central system. The building-edge monitoring unit functions not only as a data collector but also as an active component of a secure and modular disaster monitoring architecture through certificate-based authentication and local preprocessing capabilities.

### 2.3. Central Emergency Monitoring Center and Response Prioritization Mechanism

The central emergency monitoring center constitutes the decision-making and coordination layer of the proposed system architecture. As illustrated in Figure 1, this center collects and verifies data transmitted securely by building-edge monitoring units. It also generates decision-support outputs. The primary objective of the central system is to evaluate data from multiple buildings before and after disasters to generate rapid, objective response-priority outputs.

**Figure 4:**
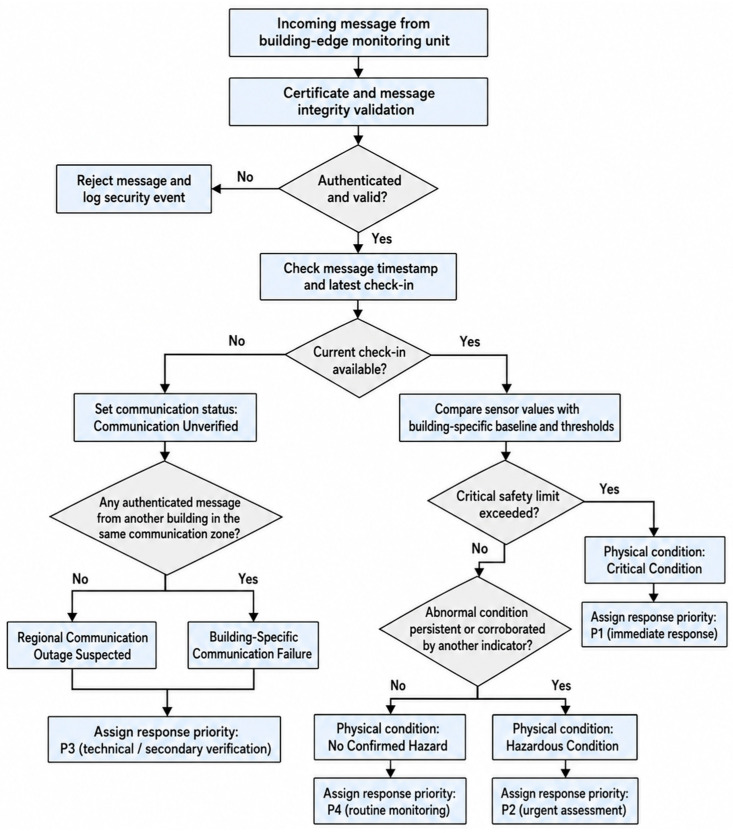
Rule-based decision workflow of the central monitoring system.

The first function of the central system is the aggregation and validation of data from building-edge monitoring units. The system accepts data only from building-edge monitoring units authenticated through the system’s certificate authority. Certificate-based mutual authentication is used to reject data from unauthorized devices or devices impersonating legitimate building-edge monitoring units. Figure 4 summarizes the rule-based decision workflow used by the central monitoring system, including message authentication, communication-status assessment, sensor evaluation, physical-condition classification, and response-priority assignment.

Data collection includes both event notifications and periodic status updates, referred to as check-in messages. These messages enable the central system to monitor communication continuity for each building-edge monitoring unit and support post-disaster situation assessment.

The central decision workflow comprises eight sequential stages: (1) message authentication, (2) message-freshness evaluation, (3) communication-zone correlation, (4) building-specific sensor evaluation, (5) abnormal-event confirmation, (6) physical-condition classification, (7) response-priority assignment, and (8) ordering within each priority level.

First, the digital certificate and integrity of each incoming message are validated. Messages from unauthorized or unverified devices are rejected and excluded from further processing. The message timestamp and the most recent check-in time for the relevant building-edge monitoring unit are then evaluated to determine whether the information is current.

If a scheduled check-in is missed, the unit is initially assigned the communication status “Communication Unverified.” The central system then evaluates authenticated messages received from other monitored buildings in the same predefined communication zone. If no monitored building within the zone can communicate, the zone is assigned the status “Regional Communication Outage Suspected.” If another building successfully transmits a normal or abnormal report, the noncommunicating unit is assigned the status “Building-Specific Communication Failure.” These communication states are evaluated independently of the physical building-condition classification.

For buildings with available sensor data, each measurement is compared with the baseline, normal variation range, warning threshold, and critical threshold defined in the building-specific configuration profile. The evaluation therefore depends on the building’s structural system, sensor type, sensor location, and commissioning measurements rather than on a universal fixed threshold.

Unless an independently defined critical safety limit is exceeded, a single transient abnormal reading does not result in a critical classification. The abnormal condition must either persist over a predefined observation interval or be corroborated by another relevant sensor indicator. This rule is intended to reduce the effects of temporary vibration, sensor noise, and isolated measurement errors.

Once an abnormal condition has been confirmed, the building is assigned one of four physical-condition classes: “Critical Condition,” “Hazardous Condition,” “No Confirmed Hazard,” or “Physical Condition Unknown.” “Critical Condition” indicates a confirmed critical structural or environmental hazard. “Hazardous Condition” indicates a persistent or corroborated warning-level hazard. “No Confirmed Hazard” indicates that recent authenticated measurements remain within the building-specific normal range. “Physical Condition Unknown” indicates insufficient current sensor evidence and should not be interpreted as structural collapse.

The central system subsequently assigns one of four response priorities. P1 indicates a confirmed critical structural or environmental condition requiring immediate response. P2 indicates a persistent or corroborated hazardous condition requiring urgent assessment. P3 indicates a building-specific communication failure or an unverified physical condition requiring technical verification or secondary assessment. P4 indicates that recent authenticated sensor data remain within the building-specific normal range and that routine monitoring should continue.

When multiple buildings receive the same priority level, they are ranked according to the magnitude of threshold exceedance, the number of concurrent hazards, the duration of the abnormal condition, the time elapsed since the latest authenticated message, and the number of corroborating sensor indicators. The resulting priority supports preliminary screening and resource allocation but does not replace an on-site structural or emergency assessment.

The second core function of the central emergency monitoring center is automated incident detection and alarm generation based on collected data. Structural and environmental data received from building-edge monitoring units are analyzed using building-specific thresholds, baseline-relative changes, persistence rules, and corroborating sensor indicators. The threshold set applied to each message is selected from the configuration profile of the relevant building and sensor location. A warning related to structural motion or inclination is not generated solely because a single sensor reading exceeds a generic value. Unless an independently defined critical safety limit is exceeded, an abnormal motion- or inclination-related condition must persist over a predefined observation interval or be supported by another relevant sensor indicator. This rule reduces the influence of transient vibration, sensor noise, or isolated measurement errors. The resulting classification is intended for preliminary screening and prioritization rather than a definitive engineering determination of collapse. This process enables the central system to identify incidents such as fire, flooding, gas leaks, or abnormal motion- or inclination-related conditions.

Unlike systems that rely on citizen reports or manual observation, this approach supports early incident detection. By evaluating data from multiple buildings simultaneously, the central system can assess incident intensity and spatial distribution across the monitored area.

A critical function of the central system is the determination of response priorities following large-scale disasters. This function is particularly important during large-area events, such as earthquakes, when simultaneous intervention at all affected buildings is impractical. An automated, data-driven prioritization mechanism can therefore support the allocation of limited resources.

In the proposed architecture, physical building condition and communication availability are evaluated separately. Physical-condition classifications are based on available structural and environmental sensor indicators. Communication status is represented independently using four states: “Verified,” “Communication Unverified,” “Building-Specific Communication Failure,” and “Regional Communication Outage Suspected.” After the first missed check-in, the building is provisionally assigned the status “Communication Unverified,” without generating a structural-risk classification.

The central system then evaluates the missed communication in relation to authenticated messages received from other monitored buildings within the same predefined communication zone. The current zone-correlation model assumes that all monitored buildings within a zone use the same cellular network operator. If no authenticated message is received from any monitored building in the zone during the specified correlation interval, the system assigns the zone-level status “Regional Communication Outage Suspected.” In this situation, individual buildings are not classified as damaged solely because they fail to communicate. If at least one building in the same zone successfully transmits a normal or an abnormal report, a complete zone-wide cellular network outage is considered less likely, and the noncommunicating unit is classified as having a “Building-Specific Communication Failure.” This status may indicate a failure of the building-edge monitoring unit, local power supply, modem, antenna, or local cellular connection. It therefore triggers a request for technical verification or secondary assessment rather than an automatic search-and-rescue dispatch.

These outputs support the prioritization of search-and-rescue and emergency response activities after a disaster.

The central emergency monitoring center is the primary component supporting the expansion of the proposed system architecture from individual buildings to multi-building deployments. Integrating secure data collection, automated incident detection, and response prioritization within a unified center is intended to reduce the fragmentation and delay in disaster-response decision-making. This structure extends the sensor-based edge monitoring approach into a decision-support framework that integrates early warning with strategic resource management.

### 2.4. Experimental Setup and Prototype

The feasibility of the proposed multi-layer emergency monitoring architecture was evaluated through a two-building prototype. The experimental configuration was designed to assess the integrated operation of the system components and observe the architecture’s behavior under normal, hazard, and communication-failure conditions. The prototype represented the interaction among the building-edge monitoring units, the sensor infrastructure, and the central monitoring system.

The experimental setup included two independent building instances, each represented by its own sensor configuration and building-edge monitoring unit. Both building-edge monitoring units communicated concurrently with the same central emergency monitoring system through independent authenticated sessions. Motion- and inclination-related structural indicators were evaluated at the building level, whereas environmental hazards, including fire, flooding, and gas leakage, were represented at both building and apartment levels. The two-building configuration was used to verify that the central system could receive, authenticate, and differentiate data from independent building-edge monitoring units. Figure 2 illustrates the representative sensor placement and connectivity within a single monitored building; the same arrangement was implemented independently for each prototype building. Table 1 summarizes the hardware, software, communication, and security components used in the prototype. In the prototype, the MPU6050 was used primarily to detect motion- and inclination-related changes, whereas the GPS module was used to provide the reference location and coarse elevation context associated with the monitored building. The fire/smoke, gas, and flood sensors were used to detect the corresponding environmental hazards and generate event notifications when the predefined experimental trigger values were exceeded.

The two buildings were not treated as comparative experimental groups. They were implemented as independent logical instances of the proposed architecture to verify concurrent authenticated communication, separate data aggregation, and correct source identification by the central system. Accordingly, the prototype evaluation focused on architectural integration and functional interaction rather than comparative analysis of building-specific event sequences. Each building-edge monitoring unit transmitted a compact TLS-protected status message at 30 s intervals. The transmission interval was calculated relative to the boot time of each controller and was not aligned with fixed wall-clock boundaries, such as the 0 and 30 s of each minute. Consequently, units booted at different times generated offset transmission schedules, reducing the concentration of periodic requests at any single instant.

For *N* building-edge monitoring units, this communication model yields an average routine request rate of *N*/*30* requests per second. As an illustrative traffic estimate, a server capable of processing 1000 HTTPS requests per second could theoretically receive periodic status messages from approximately 30,000 building-edge monitoring units. This value is a conservative analytical example rather than an experimentally measured capacity of the implemented system. An official NGINX performance evaluation reported 1735 newly established HTTPS connections per second using a four-CPU configuration [18]. However, the end-to-end capacity of the proposed system also depends on certificate-validation overhead, application-level processing, database operations, message size, network latency, abnormal-event traffic, and possible synchronized reconnection following regional power or communication outages.

The sensors assigned to each building were connected to its corresponding building-edge monitoring unit. Apartment- and building-level sensor data were locally aggregated into a single secure data stream for each building. Consequently, the two building-edge monitoring units generated separate authenticated data streams, which the central monitoring system processed concurrently. Data obtained from apartment-level sensors were not transmitted directly to the central system; instead, they were aggregated within the building-edge monitoring unit to form a single secure data stream.

In the prototype, each building-edge monitoring unit collected sensor data, performed basic preprocessing, and transmitted processed data to the central system. Each building-edge monitoring unit periodically acquired sensor data and evaluated them using building-specific baselines and trigger settings. When an abnormal condition was detected, the corresponding event information was separated from regular status updates (check-in messages) and transmitted to the central system.

In the prototype experiments, experimental trigger values were selected to activate controlled scenario events and to verify the data acquisition, transmission, and central classification workflow. These experimental trigger values were not intended to represent validated structural safety limits for a particular building type. The prototype therefore evaluates the functional implementation of the rule-based workflow rather than the engineering validity of numerical deformation limits. No universal numerical trigger set, fixed persistence duration, or common warning-to-critical transition rule was defined for the proposed architecture. In the prototype, trigger settings were configured according to the sensor type and the controlled test scenario solely to verify state transitions and message generation. In an operational deployment, these parameters would require building-specific definition and validation by qualified engineering experts.

Figure 1 illustrates the logical role of the building-edge monitoring unit and its relationship with the sensors. This architecture facilitates the independent coordination of multiple sensors within each monitored building while enabling the central system to aggregate data from multiple buildings.

In the experimental configuration, the building-edge monitoring units communicated with the central system over a cellular network. Communication was encrypted, and messages were authenticated using certificate-based authentication mechanisms. In the prototype system, only the building-edge monitoring units presenting valid certificates issued by the dedicated certificate authority were permitted to transmit data to the central system. Cellular communication was provided by a SIM7600G-H module operating over LTE/4G. A commercial mobile data service was used during the experiments. Tests were performed under normal indoor network coverage conditions. Quantitative radio-quality indicators, including RSRP, RSRQ, RSSI, and SINR, were not recorded because the study did not aim to characterize cellular-network performance. Therefore, the reported latency and power-consumption values are specific to the tested modem, cellular technology, operator infrastructure, and uninstrumented indoor coverage conditions.

This configuration reduces the risk of unauthorized devices impersonating legitimate building-edge monitoring units. The experimental setup demonstrated the functional implementability of the proposed security approach.

The prototype underwent scenario-based functional checks to observe its behavior under selected hazard and communication-failure conditions. These scenarios included changes in motion- and inclination-related structural indicators, simulated environmental hazards, and interruptions in communication between the building-edge monitoring unit and the central system.

Communication-loss scenarios were evaluated without interpreting missing communication as evidence of structural damage. When a check-in was missed, the relevant building-edge monitoring unit was initially assigned the status “Communication Unverified.” The central system then checked for an authenticated normal or abnormal report from the other building within the same communication zone. Continued communication from the other building was used to distinguish a possible building-specific failure from a zone-wide cellular network outage. In both cases, communication status was evaluated separately from the sensor-derived physical-condition classification.

### 2.5. Evaluation of the Experimental Setup

The two-building prototype demonstrated the functional feasibility of the proposed architecture and confirmed the integrated operation of the edge, communication, and central decision-making layers. The prototype integrated building-level sensor data, secure transmission to the central system, and the automated evaluation of the received information. However, the implemented setup provides proof-of-concept evidence of the conceptual architecture, whereas large-scale field performance requires further evaluation.

Although the two-building setup demonstrated simultaneous transmission and central aggregation of data from independent building-edge monitoring units, it did not reproduce the sensor density, request volume, event concurrency, or infrastructure heterogeneity expected at neighborhood, district, or regional scales. Therefore, the experimental findings should be interpreted as proof-of-concept evidence of architectural integration rather than as direct validation of regional-scale performance.

In addition to the scenario-based functional checks, end-to-end latency and building-edge monitoring unit power consumption were measured. End-to-end latency was defined as the elapsed time from detection of a predefined sensor trigger by the building-edge monitoring unit to generation of the corresponding event classification by the central system. Before timestamp recording, the clocks of the building-edge monitoring unit and central server were synchronized using the Network Time Protocol through the pool.ntp.org service. The latency measurement was repeated 100 times using the same cellular communication configuration as the prototype. The mean end-to-end latency from sensor-trigger detection at the building-edge monitoring unit to central classification was 1.28 ± 0.48 s, reported as the mean ± standard deviation for successfully completed event-classification cycles. TLS session-establishment overhead was not measured separately; therefore, the reported latency represents the overall sensor-to-classification workflow of the implemented prototype.

Power consumption was evaluated in two operating states: normal sensor monitoring and periodic check-in transmission. At a 5 V supply, the mean current consumption of one building-edge monitoring unit was 115 mA during normal monitoring and 360 mA during periodic check-in transmission. These measurements apply only to the tested prototype configuration and should not be generalized to units using different sensors, modems, microcontrollers, or communication conditions. The reported current values were measured on the 5 V load-side supply rail and exclude conversion losses associated with a future 12 V backup-battery and voltage-regulator implementation.

## 3. Discussion

This study proposed a multi-layer system architecture that integrates sensor-based building-edge monitoring units operating at the building and apartment levels with a central emergency monitoring center. The feasibility of this architecture was demonstrated through a two-building proof of concept. This section compares the proposed approach with existing disaster monitoring and response methods and discusses its advantages and limitations.

### 3.1. Comparison with Current Approaches

Conventional disaster management systems rely largely on citizen reports, manual on-site observations, and interagency communication. These conventional approaches may cause delays in information flow, misdirection, and inefficient use of response resources, particularly during large-scale disasters. In contrast, the proposed architecture uses a sensor-based infrastructure that continuously collects data and supports automated event detection without relying solely on human reporting.

Research on early warning systems and building monitoring has often focused on individual buildings or specific types of sensors. These studies have typically evaluated sensor data in isolation, limiting their applicability to multi-building decision support. In this study, however, the integration of building- and apartment-level sensor data at the edge layer and their analysis by a central system support centralized decision-making across multiple buildings. The proposed approach addresses both early warning and post-disaster response planning.

Recent IoT-based studies have demonstrated real-time vibration acquisition, low-cost damage monitoring, and remote structural assessment in laboratory structures or specific infrastructure elements [15,16]. Edge-enabled approaches have shown that local signal processing can reduce communication load and improve the timeliness of structural monitoring activities [6,7]. Table 2 compares representative related approaches with the proposed architecture in terms of monitoring scope, edge processing, security, communication-status assessment, multi-building capability, and response prioritization. The proposed architecture differs from these approaches by integrating structural and environmental sensing, certificate-based device authentication, communication-status evaluation, and central response prioritization within a unified framework demonstrated through a two-building prototype. The novelty of this study is therefore architectural and system-level rather than algorithmic. Its contribution lies in defining and implementing the interactions among multi-hazard sensing, secure device authentication, communication-status differentiation, physical-condition assessment, and centralized response prioritization within a unified multi-building framework.

As shown in Table 2, the reviewed studies primarily focus on structural monitoring and, in some cases, local edge processing. In contrast, the proposed architecture integrates structural and environmental sensing, certificate-based mutual authentication, explicit communication-status assessment, multi-building data aggregation, and centralized response prioritization. The contribution is therefore architectural and system-level rather than algorithmic.

### 3.2. Architectural Advantages

A primary advantage of the proposed architecture is the clear separation of sensing, communication, and decision-making functions. Data collection and limited preprocessing at the edge layer reduce the load on the communication infrastructure and support future system expansion. This configuration is easier to manage than architectures in which numerous sensors connect directly to the central system.

Cellular communication provides an alternative data-transmission path when fixed infrastructure is unavailable. When fixed infrastructure is damaged, cellular networks may support asynchronous data transmission and reduce the risk of a complete system failure, although congestion, power loss, or infrastructure damage may still interrupt service. In addition, certificate-based authentication and a dedicated certificate authority reduce the risks of fraudulent data injection and unauthorized device access.

The current implementation differs from more advanced edge-intelligence systems. Its local processing functions rely on transparent rules and building-specific configurations rather than learned models. This approach was selected because it is computationally lightweight, interpretable, and suitable for resource-constrained building-edge monitoring units. However, it does not support adaptive learning, pattern recognition, or collaborative inference across building-edge monitoring units.

### 3.3. Assessment in Terms of Intervention Prioritization

A key contribution of this study is the conceptual framework proposed for the automated prioritization of post-disaster responses based on sensor data and communication continuity. The central system separately assesses physical condition, communication status, and response priority to support the allocation of limited response resources.

A communication failure is not treated as an independent indicator of structural collapse or as an automatic basis for prioritizing search-and-rescue deployment. Instead, communication availability is represented as a separate status variable and evaluated in relation to authenticated reports from other buildings in the same communication zone. A building-specific communication failure triggers a request for technical verification or secondary assessment, whereas a suspected regional communication outage prevents building-level damage from being inferred solely from communication loss. This separation reduces the risk of directing scarce response resources to cases incorrectly classified as physical hazards.

The current central decision-making workflow is intentionally rule-based and interpretable; it should not be considered a formal optimization model or an intelligent risk-assessment system. Developing a reliable data-driven decision mechanism would require large-scale, multi-building datasets representing different structural systems, sensor configurations, hazard types, communication conditions, and verified incident outcomes. Such development would also require institutional approvals, coordination with emergency-management authorities, expert-defined labels, and validation against structural-engineering and operational-response criteria. Future studies may investigate formal risk models, optimization-based prioritization, and machine-learning-supported decision mechanisms once sufficiently diverse and validated field data become available.

### 3.4. Limitations

The architecture and prototype presented in this study demonstrate the feasibility of the conceptual approach but remain subject to several limitations. The experimental setup included two independent building instances; however, different building types, substantially higher sensor densities, and neighborhood-, district-, or regional-scale deployments were not experimentally tested. Therefore, the prototype demonstrates concurrent operation and central aggregation from two buildings but does not establish the operational capacity of the central system at larger scales. Although controlled server tests indicated that more than 1000 requests could be handled, this result should be interpreted as evidence of technical feasibility rather than full validation of large-scale field performance. The request-rate calculation presented in this study is an analytical estimate based on periodic 30 s status messages and should not be interpreted as an end-to-end performance benchmark. Actual system capacity may be affected by certificate validation, database transactions, application-level processing, message size, simultaneous emergency notifications, network conditions, and synchronized reconnection after widespread restoration of power or communication. Controlled load testing using large numbers of emulated building-edge monitoring units is therefore required before regional-scale capacity can be confirmed. In addition, the current evaluation does not reproduce the operational heterogeneity expected in a realistic large-scale deployment. Buildings may differ in structural system, sensor density and placement, cellular signal quality, power availability, and local network conditions. Large-scale disaster scenarios may also generate simultaneous abnormal-event messages, synchronized reconnection attempts, and temporary failures in communication or power infrastructure. Therefore, the current two-building prototype should be interpreted as a functional proof of concept rather than as evidence of operational effectiveness at the district or regional scale.

Although the revised zone-correlation algorithm reduces the risk of interpreting cellular-network outages as evidence of building damage, it cannot identify every possible cellular-network failure pattern. For example, buildings located near one another may occasionally connect through different base stations, whereas a single base-station failure may affect only part of a predefined zone. Therefore, a valid message from another building reduces the likelihood of a complete regional outage but does not confirm the physical condition of the noncommunicating building. Communication-derived statuses therefore remain separate from sensor-derived physical-condition classifications, and communication loss without corroborating sensor evidence requires technical verification or secondary assessment.

The proposed security model also has residual limitations. Mutual TLS, device-specific certificates, certificate revocation, timestamps, and duplicate-message checks reduce the risks of device impersonation, unauthorized transmission, message alteration, and replay attacks but do not eliminate all possible threats. The current prototype does not fully address physical capture of a building-edge monitoring unit, extraction of private keys from inadequately protected hardware, compromise of the certificate authority, insider threats, denial-of-service attacks, malicious firmware modification, or prolonged connectivity loss. Operational deployment would therefore require hardware-backed key storage, protected certificate-authority infrastructure, secure firmware-update mechanisms, rate limiting, intrusion monitoring, service redundancy, and periodic security audits.

The prototype was evaluated under laboratory conditions and was not subjected to controlled environmental qualification testing. Although commercially available development-board sensors were sufficient to demonstrate data-acquisition and event-generation functions, their precision and stability under elevated temperature, humidity, vibration, shock, dust, water exposure, or prolonged power interruption were not evaluated. Consequently, the current prototype should not be considered operationally ready for deployment under real disaster conditions. An operational implementation would require industrial-grade sensors, protective enclosures, backup power, calibration procedures, and environmental testing appropriate to the intended deployment conditions.

The study does not derive or validate numerical structural-safety thresholds for specific building types. The values used in the prototype were experimental triggers intended to verify the functional workflow. Operational deployment would require building-specific baseline establishment and the definition of warning and critical limits by qualified structural engineers. These limits should account for the structural system, number of stories, sensor location, baseline response, and applicable engineering standards. Therefore, the current prototype should not be used as a substitute for a formal structural-safety assessment.

The P1–P4 prioritization workflow was functionally implemented in the prototype but was not validated using a comprehensive test-case matrix covering transient noise, conflicting sensor inputs, simultaneous hazards, missed check-ins, and regional communication failures. Therefore, the current workflow should be interpreted as a rule-based proof-of-concept decision-support mechanism rather than as a validated operational prioritization model.

Large-scale field validation was beyond the scope of this study because deployment across numerous buildings would require extensive hardware installation, institutional approvals, infrastructure access, and coordination with local authorities.

### 3.5. Forward-Looking Assessment

Despite these limitations, the proposed architecture provides a basis for secure, sensor-based, and centralized decision-support systems in disaster management. The system’s effectiveness may be improved through larger-scale field deployments, disaster-specific assessment models, and advanced data analytics methods. The study provides a foundation for a comprehensive approach spanning pre-disaster early warning and post-disaster response planning.

## 4. Conclusions and Future Work

This study proposes a multi-layer disaster-monitoring and decision-support architecture. This architecture integrates a central emergency monitoring center with sensor-based building-edge monitoring units deployed at both the building and apartment levels. The proposed approach integrates pre-disaster early detection, real-time monitoring during disasters, and post-disaster response prioritization within a single architectural framework. Unlike approaches based primarily on citizen reports, the system supports objective and automated assessment through a sensor infrastructure that continuously collects data.

The architecture provides a structured and manageable framework by clearly separating the edge-sensing, secure-communication, and central decision-making layers. The building-edge monitoring unit reduces communication load by aggregating data from multiple sensors and limiting the number of direct connections to the central system. The LTE/4G communication layer provides a cellular data path, although uninterrupted connectivity cannot be guaranteed during disasters. Certificate-based authentication and a dedicated certificate authority reduce security risks, including unauthorized device access and fraudulent data injection. However, the experimental evidence is limited to a two-building prototype, and system capacity at neighborhood, district, or regional levels has not yet been experimentally validated.

The proposed central emergency monitoring center functions as both an incident-monitoring system and a decision-support component for post-disaster response prioritization. The separate assessment of physical condition, communication status, and response priority based on sensor data and communication continuity supports the allocation of limited response resources. The study demonstrates the functional feasibility of integrating sensor-based disaster-monitoring systems with disaster management processes.

However, the study used a limited two-building prototype to demonstrate the feasibility of the architecture. Further studies are required to evaluate different building types, sensor densities, and large-scale distributed deployments. Factors such as sensor reliability, environmental effects, and regional communication-infrastructure outages may affect system performance.

Future studies may extend the indoor sensing capabilities of the proposed architecture through camera-based systems. For elderly individuals living alone, artificial intelligence-based analysis of camera data may support the rapid and automated identification of emergency situations. Events such as the absence of a person from the camera’s field of view for a predefined period or prolonged immobility after a fall could be transmitted to the central system as additional risk indicators. This approach may improve emergency-response decisions by integrating sensor-based data with visual context. Any future camera-based extension should follow privacy-by-design principles and operate locally at the building-edge layer. Image inference and event detection should be performed on the edge device, without transmitting raw video or still images to the central monitoring system. Only event-level outputs, such as detected event type, confidence score, timestamp, and device identifier, should be transmitted. Raw image retention should be disabled by default. If temporary local buffering is technically required for event verification or system validation, the retention period should be predefined, minimal, and automatically enforced. Any locally retained data should be encrypted and protected through access controls and audit logging. Implementing such a system would also require informed consent, transparent notification of residents, purpose limitation, and compliance with applicable personal-data-protection regulations. In this context, the proposed architecture may provide a flexible infrastructure for both disaster-focused applications and continuous-care and early-intervention scenarios involving vulnerable groups.

Future studies will assess the proposed architecture through larger multi-building field deployments. Disaster-specific assessment models may also be developed. Future work may also investigate advanced analytical methods that combine communication status, sensor data, and historical event records. Data-analytics and machine-learning methods may reduce false-positive classifications and improve response prioritization. The study provides a foundation for future research on secure, modular, and data-driven disaster management systems.

Hybrid fallback technologies, including LoRaWAN and satellite communication, may improve resilience where the required infrastructure, coverage, power supply, regulatory conditions, and operational budget are available. However, these alternatives require additional terminals, gateways, or service subscriptions and may not provide a universally available or cost-effective fallback option. They were therefore not incorporated into the current low-cost prototype but may be evaluated as deployment-specific extensions in future studies.

## Figures and Tables

**Table 1:** Hardware, software, communication, and security configuration of the prototype.

Component/Parameter	Prototype Configuration
Microcontroller	Arduino Uno
Cellular Module	SIM7600G-H
Tilt/Motion Sensor	MPU6050
GPS Module	NEO-6M
Fire/Smoke Sensor	HW-072
Gas Sensor	MQ-5
Flood Sensor	DFR0151
Sampling Interval	5 s
Local Preprocessing	Building-specific threshold and baseline evaluation
Data-transmission Interval	30 s
Event Transmission	Immediate transmission after detection
Communication Protocol	HTTP over TLS
Authentication	Mutual certificate-based authentication
Certificate Validation	CA chain, validity period, device identity and revocation checks
Message Fields	Device/building ID, timestamp, message ID, event type, value/status and communication flag
Server-Side Implementation Language	PHP 8.3.6
Server Operating System	Ubuntu 22.04
Web Server	NGINX 1.24.0
Database	MySQL 8.0.46
TLS Version	TLS 1.3
Certificate Format	X.509
Key Algorithm	RSA
Key Length	2048 bit
Central Server Hardware	Intel(R) Core(TM) i7-13620H
	8 GB RAM
	SSD

**Table 2:** Comparison of representative related approaches and the proposed architecture.

Study	Monitoring Scope	Edge Processing	Multi-Hazard Monitoring	Secure Device Authentication	Communication Status Assessment	Multi-Building Architecture	Response Prioritization
Lin et al. [6]	Structural health monitoring of bridges	Yes	No	Not reported	No	No	No
Hidalgo-Fort et al. [7]	Structural health monitoring using low-cost edge nodes	Yes	No	Not reported	No	No	No
Saravanan et al. [15]	Laboratory-scale structural health monitoring	Limited/local processing	No	Not reported	No	No	No
Rehman et al. [16]	Vibration-based remote structural monitoring	Yes	No	Not reported	No	No	No
Proposed architecture	Building- and apartment-level structural and environmental monitoring	Yes, rule-based	Yes	Yes, certificate-based mutual authentication	Yes	Yes	Yes

## Data Availability

The original contributions presented in this study are included in the article.
